# Exploring the interplay of stress, fatigue, and empathy: The mediating role of cognitive flexibility in enhancing the well-being of university students in medical and social disciplines

**DOI:** 10.1371/journal.pone.0321946

**Published:** 2025-04-24

**Authors:** Agata Rudnik, Krzysztof Sobczak, Artur Sawicki, Agata Zdun-Ryżewska

**Affiliations:** 1 Institute of Psychology, Faculty of Social Sciences, University of Gdańsk, Gdańsk, Poland; 2 Academical Psychological Support Centre, University of Gdańsk, Gdansk, Poland; 3 Department of Gastroenterology, Independent Public Health Care of the Ministry of the Internal Affairs, Gdansk, Poland; 4 Department of Sociology of Medicine and Medical Communication, Faculty of Health Sciences, Medical University of Gdańsk, Gdańsk, Poland; 5 Department of Quality of Life Research, Faculty of Health Sciences, Medical University of Gdańsk, Gdansk, Poland; St John's University, UNITED STATES OF AMERICA

## Abstract

This study investigates the interplay between stress, fatigue, and empathy among university students in medical and social disciplines, focusing on the mediating role of cognitive flexibility. University students, particularly those in medical fields, experience significant stress and fatigue, which adversely affect their well-being and academic performance. Empathy, essential in both interpersonal and professional contexts, is negatively impacted by stress and fatigue. Cognitive flexibility, the ability to adapt and manage diverse cognitive demands, is proposed as a key mediator that could alleviate the negative effects of stress and fatigue on empathy. The study involved 1,701 students from the Medical University of [to preserve anonymity, the institution’s name has been omitted] and the University of to preserve anonymity, the institution’s name has been omitted], who completed validated questionnaires measuring perceived stress, fatigue, empathy, and cognitive flexibility. The results indicated that cognitive flexibility was positively related to empathy and negatively related to stress and fatigue. Mediation analyses revealed that cognitive flexibility significantly mediated the relationships between stress and empathy, as well as between fatigue and empathy. These findings suggest that enhancing cognitive flexibility may help students better manage stress and fatigue, thereby maintaining higher levels of empathy. This research contributes to understanding the cognitive and emotional interactions in educational settings and highlights the importance of developing interventions that enhance cognitive flexibility to support student mental health and academic success.

## Introduction

The college years are notably stressful for many students, who frequently encounter a myriad of challenges that can impact their psychological well-being and academic performance [1]. This study aims to explore constructs like stress, fatigue, and empathy within the context of cognitive flexibility, which may play a mediating role in how these factors interrelate.

University students experience a high prevalence of mental health issues, including stress and fatigue [2–5]. Moreover, studies in medical education indicate that medical students experience higher levels of mental distress compared to the general population [6].

Stress among students is widely recognized as a major concern in educational research due to its impact on learning, well-being, and overall development [7,8]. Academic demands, social pressures, and the transition to independence are common sources of stress in student populations. Research has shown that high levels of stress can lead to decreased academic performance and increased vulnerability to psychological issues such as anxiety and depression [9,10].

Fatigue in students can be both a consequence of prolonged stress and a contributor to decreased academic and personal efficacy. It is often reported as a common issue that affects concentration, motivation, and academic engagement [11,12]. Understanding how fatigue interacts with other psychological factors like stress and empathy is crucial for developing effective educational and wellness programs.

Empathy is defined as the ability to recognize and respond to other people’s thoughts, intentions, desires, and emotions with appropriate emotional reactions [13,14]. It serves a vital role in both interpersonal and societal contexts by facilitating the sharing of experiences, needs, and desires among individuals. Empathy acts as an emotional bridge that fosters prosocial behaviour, enhancing cooperation and understanding within communities [14]. Therefore, empathy is crucial in professions where effective communication is essential. Identifying factors related to empathy is important for understanding how to teach empathetic behaviour [15,16]. However, significant evidence indicates a decline in empathy levels among medical students over the years [17,18]. While empathy can enrich the educational experience, it also requires emotional resources that may be depleted by stress and fatigue, potentially diminishing a student’s ability to engage empathetically [19]. This underscores the importance of psychological resources, including those that enable adaptation to the various challenges that studying brings.

Cognitive flexibility is crucial for adapting to continuously changing environments and is associated with various goal-oriented behaviours such as creativity, problem-solving, multitasking, and decision-making, although its concepts have been challenging to define [20,21]. For this study, we adopted the definition of cognitive flexibility from Dennis and Vander Wal [22], as outlined in the Cognitive Flexibility Inventory (CFI). This definition emphasizes the type of cognitive flexibility necessary for individuals to effectively challenge and replace maladaptive thoughts with more balanced and adaptive thinking. It is measured in three areas: (a) the inclination to view challenging situations as controllable, (b) the capability to recognize multiple alternative explanations for life events and human behaviour, and (c) the skill to devise various alternative solutions to difficult situations.

While previous studies have examined the individual effects of stress, empathy, fatigue, and cognitive flexibility in educational settings, there is a paucity of research exploring how these factors might interact dynamically. Specifically, the potential mediating role of cognitive flexibility in the relationship between stress, empathy, and fatigue has not been thoroughly investigated among student populations.

This study seeks to fill this gap by examining these relationships within a diverse student sample. This research is significant as it addresses critical elements of student well-being and academic success. By exploring the mediating role of cognitive flexibility, the study may offer insights into new strategies for educational support services to enhance student resilience against stress and fatigue while promoting empathetic engagement. The findings could contribute to theoretical advancements in understanding cognitive and emotional interactions in learning environments, potentially informing the development of targeted interventions that support student mental health and academic achievement.

## Materials and methods

The recruitment process took place from April 3rd, 2019, to January 31st, 2020. A total of 1,783 students from the Medical University of [to preserve anonymity, the institution’s name has been omitted] and the University of [to preserve anonymity, the institution’s name has been omitted] took part in the study. However, 82 participants did not complete the questionnaire fully, leading to their exclusion from the final analysis. Consequently, 1,701 completed questionnaires were analysed, representing 95.4% of the returned questionnaires. The final sample included 1,001 students (N=1001) enrolled in medical-related programs and 700 students (N=700) from non-medical disciplines. It should be noted that there were discrepancies in tests due to missing data (N ranges from 1,670–1,690). The socio-demographic characteristics of the participants are detailed in Table 1.

**Table 1:** Socio-demographic characteristics of the participants.

	Students (N; %)
Age (in years)	
18-20	634 (37%)
21-23	825 (49%)
24 and above	236 (14%)
Gender	
Female	1366 (81%)
Male	319 (19%)
Type of University	
Medical University	1001 (59%)
University	700 (41%)
Years of Studies	
First-year	472 (28%)
Second-year	323 (19%)
Third-year	292 (17%)
Fourth-year	311 (18%)
Fifth-year	297 (18%)

### Methods

Stress levels were measured using the Perceived Stress Scale (PSS-10) developed by Cohen, Kamarck, and Mermelstein in 1983 [23]. This 10-item tool is designed to measure overall levels of perceived stress over the past month. It has been scientifically adapted with verified and confirmed reliability and validity by Juczyński and Ogińska-Bulik in 2009 [24], and is extensively used throughout Poland for scientific research and psychological assessment.

To assess the mental and physical dimensions of fatigue, we utilized the 11-item Chalder Fatigue Questionnaire (CFQ) as developed by Cella and Chalder in 2010 [25]. The Polish version of this instrument adapted by Zdun-Ryzewska, Basiński and Michalik in 2020 [26] has been shown to be as reliable and valid as the original version.

The Empathy Quotient (EQ) questionnaire, developed by Baron-Cohen and Wheelwright in 2004 [27] and validated in Polish by Jankowiak-Siuda et al. in 2017 [28], was also employed. The EQ-40 test includes 40 diagnostic items rated on a four-point Likert scale, designed to assess empathy in individuals with normal intelligence levels [29].

Cognitive flexibility was assessed using the Cognitive Flexibility Inventory (CFI), designed by Dennis & Vander Wal in 2010 [22], with a Polish adaptation developed by Piórowski et al. in 2017 [30]. This brief self-report consists of 20 statements where respondents select one of the possible responses, ranging from 1 (“definitely disagree”) to 7 (“definitely agree”).

Additionally, a supplementary section with 10 questions captured demographic and social information about the participants, such as age, gender, year and field of study, mode of learning, specialization, and employment status during education. These questions served as independent variables.

All statistical analyses were conducted using the IBM SPSS Amos 27.0 software package.

### Ethics statements

The project was carried out under a bilateral agreement between the universities and received approval from the Ethics Committee for Research Projects at the Institute of Psychology, University of [to preserve anonymity, the institution’s name has been omitted] (Approval No. 4/2019). Students who provided their written consent were invited to participate in the study after their course classes. Participation was voluntary and anonymity was preserved. The data were analysed anonymously.

## Results

Means, standard deviations and Pearson’s *r* correlation coefficients are presented in Table 2. Cognitive flexibility was negatively related to stress and fatigue, and positively related to empathy. Moreover, fatigue was negatively related to empathy, and positively with stress. Stress was not related to empathy.

**Table 2:** Means, standard deviations, and correlation coefficients between studied variables.

Variable	*M*	*SD*	1	2	3
1. Fatigue	15.85	6.01			
2. Stress	19.96	9.26	.36^**^		
3. Cognitive flexibility	103.14	15.54	−.18^**^	−.28^**^	
4. Empathy	43.73	10.00	−.07^*^	−.05	.37^**^

*Note. N* = 1701. It differentiates in tests due to missing data (*n* = 1670–1690).

* *p* <.01;

** *p* <.001.

**Fig 1:**
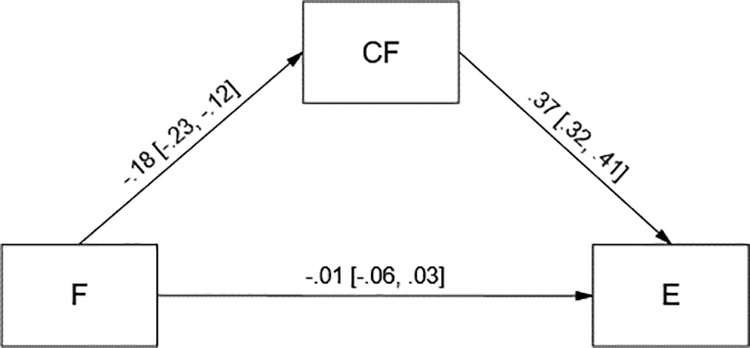
Tested model in which cognitive flexibility (CF) mediated the relationship between fatigue (F) and empathy (E). Standardized coefficients with 95% bias-corrected bootstrap confidence intervals are presented. Indirect effect of fatigue on empathy was −.07 [−.09, −.04]. **n** = 1661. Forty participants were excluded from the analyses due to missing data.

**Fig 2:**
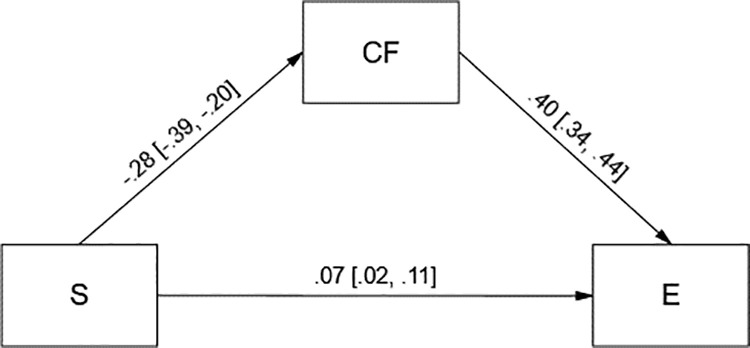
Tested model in which cognitive flexibility (CF) mediated the relationship between stress (S) and empathy (E). Standardized coefficients with 95% bias-corrected bootstrap confidence intervals are presented. Indirect effect of fatigue on empathy was −.11 [−.16, −.08]. **n** = 1679. Twenty-two participants were excluded from the analyses due to missing data.

Assumed mediational effects were tested using 95% bias-corrected confidence interval (95% CI) bootstrap method with 5000 samples. Both models were saturated (*df* = 0), therefore we do not report standard fit indices. Cognitive flexibility (CF) significantly mediated the relationship between fatigue (F) and empathy (E) (Fig 1). It also significantly mediated the relationship between stress (S) and empathy (Fig 2). In both models cognitive flexibility was directly and positively related to empathy.

## Discussion

This study underscores the pivotal role of cognitive flexibility in mediating the relationships between stress, fatigue, and empathy among university students, particularly those in medical and social disciplines. By elucidating these relationships, the findings provide a nuanced understanding of the cognitive and emotional dynamics that underpin well-being and academic performance in higher education. Below, we discuss the implications of these findings in greater detail, integrating relevant literature for a comprehensive perspective.

University students frequently experience stress and fatigue, which adversely affect their health, academic performance, and social adaptation [31]. Consistent with prior findings, our study revealed a positive correlation between stress and fatigue. Walkiewicz et al. (2023) highlighted that fatigue among medical students often results from high academic demands and the stress inherent in medical education [32]. These challenges can diminish quality of life, reduce satisfaction with studies, and exacerbate stress levels. However, participants in our study with higher cognitive flexibility reported lower levels of stress and fatigue.

The analysis by Kruczek, Basinska, & Janicka (2020) demonstrated that cognitive flexibility enables nurses to employ a broader range of coping strategies effectively [33]. This adaptability not only aids in managing personal stress but also enhances patient care and reduces errors in clinical practice. Similarly, in our study, cognitive flexibility emerged as a significant mediator, buffering the negative effects of stress and fatigue on empathy. This finding aligns with Alsaif et al. (2024), who proposed that cognitive flexibility facilitates adaptive responses to changing environments, helping students manage academic stressors while maintaining empathetic engagement [34]. Such adaptability is particularly critical for students in high-stress fields like medical training, where emotional resilience is essential for professional success. Furthermore, research highlights that cognitive flexibility enhances student engagement [35] and serves as a protective factor against the adverse effects of stress in educators [36]. This capacity allows students to reframe maladaptive thoughts and develop balanced responses, ultimately supporting personal and academic growth.

Empathy, a cornerstone of both medical and socially oriented professions, was negatively correlated with fatigue and stress, and positively correlated with cognitive flexibility in our analyses. Studies consistently demonstrate that higher stress levels are associated with reduced empathy among students, as the emotional and cognitive demands of stress can lead to burnout and diminish empathetic engagement [37–39]. Cognitive flexibility, however, acts as a protective factor. Cai & Qi (2023) described its multifaceted role in enhancing emotional regulation, modifying empathetic engagement, and buffering against the cognitive and emotional demands of empathy [40]. This dual role underscores the intricate interplay between cognitive processes and emotional experiences in social interactions.

In our study, cognitive flexibility significantly mediated the relationships between both fatigue and empathy, and stress and empathy. It was positively associated with empathy in both cases, reinforcing its protective role. According to Yu, Yu, & Lin (2019), cognitive flexibility plays a pivotal role in moderating impulsivity, particularly in individuals experiencing anxiety and depression [41]. This adaptive mental capability enables individuals to shift thoughts and responses according to external demands, facilitating better decision-making and behavioral responses. Additionally, cognitive flexibility appears crucial for adapting to changing social norms and personal habits, especially in the context of smartphone and social media overuse. Enhancing this cognitive ability may mitigate the negative social and psychological impacts of technology dependence [42].

### Implications and recommendations

The results of this study have significant implications for educational practice, societal well-being, and future scholarship.

In educational contexts, cognitive flexibility training should be integrated into curricula through workshops or courses that employ evidence-based techniques such as mindfulness, cognitive-behavioural strategies, and adaptive thinking exercises. For example, scenario-based learning can help prepare students for high-stress, empathy-dependent situations in their respective fields. Tailored interventions are essential to address the unique needs of students in medical and social science disciplines. Medical students, for instance, could benefit from empathy simulation programs that replicate patient interactions, while social science students may gain valuable skills through community-based experiential learning programs. Additionally, mentorship programs that pair experienced students with newcomers can promote the exchange of strategies for managing stress and enhancing cognitive flexibility, thereby fostering a supportive academic environment. Universities should also invest in mental health resources and cultivate an environment that prioritizes student well-being, particularly during high-stress periods such as exams or clinical rotations.

On a societal level, public awareness campaigns should emphasize the importance of cognitive flexibility and stress management, particularly for young adults entering high-stakes professions. These campaigns could involve collaboration with mental health organizations to distribute resources and tools aimed at building resilience. Community engagement initiatives, such as volunteer programs, offer students opportunities to practice empathy in real-world settings, thereby enhancing their social and emotional competencies while contributing to societal well-being.

For scholarship, this study opens avenues for longitudinal research to explore causal relationships between stress, fatigue, empathy, and cognitive flexibility over time. Such studies could provide deeper insights into how these constructs interact and evolve, informing the design of more effective interventions. Additionally, neuroscientific exploration of the mechanisms underlying cognitive flexibility and its role in emotional regulation could offer targeted evidence for practical interventions.

By addressing these implications, this research not only enriches theoretical understanding but also provides actionable strategies for enhancing student well-being and professional readiness. Future studies should aim to validate these findings across diverse cultural contexts, ensuring that interventions are inclusive and globally relevant.

### Limitations

The findings of this study should be interpreted in light of several limitations. First, the sample was predominantly female (81%), which reflects the typical gender demographics of the selected academic institutions but limits the generalizability of the results, particularly concerning cognitive flexibility and empathy among male students. Additionally, while the study employed validated tools with global applicability, the findings may still be influenced by cultural norms specific to Poland. Future research conducted in more diverse cultural settings would help establish the universality of these results.

The cross-sectional design of the study precludes any causal conclusions about the relationships between stress, fatigue, empathy, and cognitive flexibility. Although the mediation models suggest potential pathways, longitudinal studies are necessary to confirm causality and the stability of these relationships over time. Furthermore, the reliance on self-report questionnaires introduces the possibility of social desirability bias and inaccuracies in self-perception, which could be mitigated in future studies through the use of objective measures or peer-reports.

The categorization of participants into medical and non-medical students did not account for the specific disciplines within these broader categories, which may have unique stressors and coping mechanisms. Future research should explore such discipline-specific nuances to provide a more detailed understanding. Moreover, potential confounding factors, such as socioeconomic status, academic workload, access to mental health resources, and personal life circumstances, were not explicitly controlled and may have influenced the observed relationships.

Although cognitive flexibility was examined as a mediator, other potential mediators (e.g., emotional regulation, resilience) and moderators (e.g., support systems, personality traits) were not included in this study. Examining these factors could offer a more comprehensive understanding of the interplay between stress, fatigue, and empathy. Finally, while the study emphasizes the importance of cognitive flexibility, specific interventions to enhance this skill were not tested. Future research could focus on evaluating the effectiveness of tailored interventions aimed at improving cognitive flexibility, student well-being, and empathetic engagement.

## Conclusions

This study addresses an important gap in the literature by exploring the mediating role of cognitive flexibility in the relationships between stress, fatigue, and empathy among university students in medical and social disciplines. Previous research primarily examined these factors in isolation, without accounting for their dynamic interplay or the protective role of cognitive flexibility. Our findings underscore the critical importance of cognitive flexibility as a buffer against the negative impacts of stress and fatigue on empathy, aligning with prior studies that highlight its role in emotional regulation and adaptive coping strategies. By identifying cognitive flexibility as a key mediator, this research advances theoretical understanding of the cognitive-emotional interactions within educational contexts and provides practical insights for developing interventions tailored to students’ specific needs. The results emphasize the need for educational strategies that foster cognitive flexibility to enhance students’ ability to manage stress and fatigue effectively. Such interventions could promote better empathetic engagement, which is particularly critical in professions requiring strong interpersonal skills. For instance, integrating cognitive flexibility training into university curricula or offering targeted workshops could equip students with tools to better navigate academic and interpersonal challenges. Additionally, mental health services on campuses might consider incorporating cognitive flexibility assessments and tailored support strategies to address individual student needs.

Our findings also provide valuable implications for addressing empathy decline among medical and social discipline students, a concern well-documented in previous literature. Empathy is critical for successful interpersonal communication and professional performance, particularly in medical and helping professions. Enhancing cognitive flexibility may help students maintain higher empathy levels, even in high-stress situations, thereby improving both their academic and professional outcomes.

Future research should extend these findings by exploring the underlying neural mechanisms that link cognitive flexibility with empathy, stress, and fatigue. Longitudinal designs could provide deeper insights into how these relationships evolve over time and whether interventions can sustain long-term benefits. Additionally, expanding research to include more diverse student populations and educational settings would enhance the generalizability of the results. Ultimately, this study highlights the critical role of cognitive flexibility in mitigating the adverse effects of stress and fatigue on empathy among university students. By doing so, it opens new avenues for developing educational and mental health interventions that prioritize holistic student well-being and foster adaptive, empathetic professionals for the future.

## Supporting information

S1 File Data base. (XLS)
